# Opposing Trends in Total Knee and Hip Arthroplasties for Patients With Rheumatoid Arthritis vs. the General Population—A 14-Year Retrospective Study in Taiwan

**DOI:** 10.3389/fmed.2021.640275

**Published:** 2021-04-20

**Authors:** Kuan-Kai Tung, Yung-Heng Lee, Chuan-Chao Lin, Cheng-Hung Lee, Mei-Chen Lin, James Cheng-Chung Wei

**Affiliations:** ^1^Department of Orthopedics, Taichung Veterans General Hospital, Taichung, Taiwan; ^2^Department of Senior Services Industry Management, Minghsin University of Science and Technology, Hsinchu, Taiwan; ^3^Department of Recreation and Sport Management, Shu-Te University, Kaohsiung, Taiwan; ^4^Department of Orthopedics, Cishan Hospital, Ministry of Health and Welfare, Kaohsiung, Taiwan; ^5^Department of Physical Medicine and Rehabilitation, Chung Shan Medical University Hospital, Taichung, Taiwan; ^6^School of Medicine, Chung Shan Medical University, Taichung, Taiwan; ^7^Department of Food Science and Technology, Hung Kuang University, Taichung, Taiwan; ^8^Management Office for Health Data, China Medical University Hospital, Taichung, Taiwan; ^9^Department of Allergy, Immunology and Rheumatology, Chung Shan Medical University Hospital, Taichung, Taiwan; ^10^Institute of Medicine, College of Medicine, Chung Shan Medical University, Taichung, Taiwan; ^11^Graduate Institute of Integrated Medicine, China Medical University, Taichung, Taiwan

**Keywords:** rheumatoid arthritis, total knee arthroplasty, total hip arthroplasty, population-based, pharmacological influences, big data and analytics

## Abstract

**Objective:** To determine the trend of incidence rate of total knee arthroplasty (TKA), total hip arthroplasty (THA), and TKA or THA (major joint arthroplasty, MJA) among rheumatoid arthritis (RA) population and compared them with general population (GP) in Taiwan.

**Methods:** Incidence rates and trends of TKA, THA, and MJA were determined over a 14-year period (2000–2013) among RA patients and compared them with GP. RA of patients was diagnosed based on the ACR 1987 criteria and extracted from GP. Subanalyses of incidences of TKA, THA, and MJA by year, 10-year age group, and gender were further conducted for demographic analysis. Patient profiles were extracted from the National Health Insurance Research Database (NHIRD) for interrupted time-series analysis and cohort studies.

**Results:** Patients enrolled were 168,457 receiving TKA, 64,543 receiving THA, and 228,191 receiving MJA surgery. Incidences of TKA, THA, and MJA in RA patients were significantly lower by 49.0, 41.5, and 41.0% compared with concomitantly rises in GP by 131.0, 25.1, and 90.0% among the GP during the study period. The dominant age population for TKA, THA, and MJA were those aged 70–79 years in both GP and RA groups.

**Conclusions:** We found an opposing trend in incidence of TKA, THA, and MJA between RA patients and the GP. The possible influence of pharmacological treatment is implicated for the lower incidence rates of TKA, THA, and MJA surgeries among RA patients.

## Introduction

Rheumatoid arthritis (RA) is one of the most prevalent chronic inflammatory diseases causing structural changes such as major joint deformity and soft tissue damage (1). RA affects 0.1–0.3% of Asians, and it has an incidence rate of 15.8 per 100,000 people in Taiwan (2, 3). Medications for RA are to improve function and prevent persistent major joint destructions. Conventional synthetic disease-modifying antirheumatic drugs (csDMARDs) and biological DMARDs (bDMARDs), applied as monotherapy or in combination with other anti-inflammatory drugs, had showed promising efficacy in preventing RA from progressing to late stages and improving functional outcome during since late 1980s (4). In Taiwan, lower healthcare utilization cost was offset with the increase medication cost in biologics such as csDMARDs and bDMARDs since 1990s (5).

Despite the effectiveness of pharmacological treatment, orthopedic procedures remain indispensable in managing severe RA. Among these procedures, total joint arthroplasty is the only available solution for non-reversible joint destruction of these patients. RA patients in Taiwan suffer 4.02 times greater risk, compared with non-RA patients, of undergoing major joint arthroplasty (MJA) surgeries, including total knee arthroplasty (TKA) and total hip arthroplasty (THA) (6). In the last three decades, 30–50% of RA patients have undergone orthopedic procedures. Specifically, up to 24% of patients with RA underwent MJA with substantial improvements of overall function and quality of life (7, 8).

In recent years, the fewer MJA in reducing joint erosion and chronic inflammatory progress is thought to be related to increased intensity and the introduction of more effective anti-rheumatic medications, like conventional and biological DMARDs (9–12). Pharmacological treatments are recommended as first line therapy for RA patients since MJA has greater complication risks and is a more expensive procedure (13). Whereas, among the general population (GP), the growing demand for MJA has made it the most common elective surgery (14). But few studies have compared incidences of MJA between RA patients and GP among Asian populations, including those in China and Taiwan.

Therefore, we aimed to determine population-based incidence rates of TKA, THA and MJA among a well-defined RA population and in GP of Taiwan, using the National Health Insurance Research Database (NHIRD) collected between 2000 and 2013.

## Materials and Methods

### Data Source

The Taiwan National Health Insurance Research Database (NHIRD) was first established in 1995. The health information it contained included outpatients, hospitalization, prescription, surgery and other medical services derived from the single payer health insurance program for the general population. In this study, we have chosen the Taiwan population-based hospitalization files to conduct our analyses. The identification numbers in the database were already encrypted before release to protect patients' privacy. Diagnosis of diseases was based on International Classification of Diseases, Ninth Revision, Clinical Modification (ICD-9-CM). The Research Ethics Committee of China Medical University and Hospital in Taiwan approved our study [CMUH-104-REC2-115-(AR4)]. We disclaimed that there is no potentially identifiable images or data, animal, or human study involved in current study.

### Study Population

We aimed to evaluate the annual incidence and the consecutive trend of TKA (ICD-9-OP: 81.54), THA (ICD-9-OP: 81.51) and MJA (defined as patient receiving TKA or THA) among GP and patients with RA (ICD-9-CM: 714.0) in Taiwan from 2000 to 2013. RA cohort was defined as: (1) Patients with newly diagnosed RA and with catastrophic illness card from 2000 to 2012 were identified as RA patients; (2) The date of the catastrophic illness card of RA applied was set up as the index date; (3) Patients were followed from the index date to TKA, THA, MJA (TKA or THA) diagnosis, death, withdrawn from NHIRD, or the end of the year 2013 which came first. Two study populations were enrolled: GP and RA cohorts. The GP cohort consisted of almost every hospitalized patient who had received surgery of TKA (ICD-9-OP: 81.54), THA (ICD-9-OP: 81.51), or MJA in the NHIRD database regardless of underlying diagnosis. Patients in the RA cohort were a subset of the GP cohort that underwent TKA, THA, or MJA surgery under the diagnosis of RA. The diagnosis of RA, defined by catastrophic illness card during the study period, was certified by rheumatologists according to the criteria of American College of Rheumatology in 1987 (ACR 1987) (15). Patients underwent TKA, THA, or MJA before the diagnosis of RA, having incomplete information like age or gender, or recorded on December 31, 2013 were excluded from the RA cohort.

### Statistical Analyses

Demographic parameters and baseline comorbidities were included in our analysis. Age group, gender, occupation type, and the presence of baseline comorbidities were defined as categorical variable. The annual incidences of TKA, THA, and MJA by year, 10-year age group, and gender were calculated by the number of surgical events divided by total population person years (per 100,000 person years in GP and per 1,000 person years in patients with RA). The 95% confidence intervals for incidence were calculated by Fisher's exact test. Compared with the year 2000, differences in incidence rate (from 2001 to 2013) of all surgical types were evaluated by the Poisson regression model. We further verified the association between comorbidities and TKA, THA, and MJA by Cox model. Analyses were performed with the SAS 9.4 software (SAS Institute, Cary, NC, USA). Two-sided *p*-values < 0.05 were considered statistically significant.

## Results

**Figure 1:**
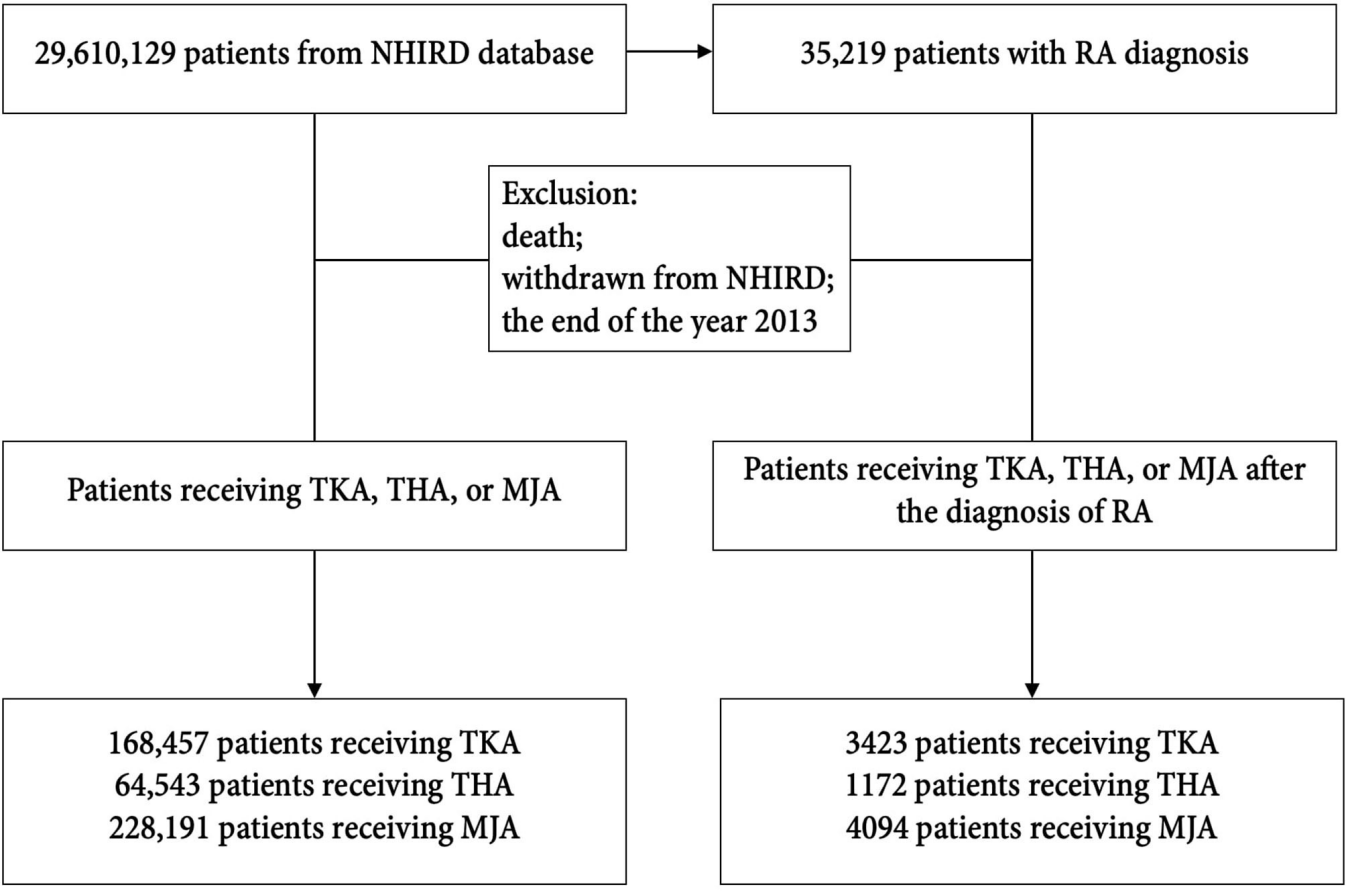
Flowchart of the study selection process.

The NHIRD data base comprised of 29,610,129 registered patients (GP cohort), including 35,219 RA patients. After applying the study eligibility criteria, we enrolled 168,457 patients receiving TKA surgery, 64,543 patients receiving THA surgery, and 228,191 patients receiving MJA surgery during the study period (from 2000 to 2013) in the GP cohort. Among those patients, 3,423 (2.03%), 1,172 (1.81%), and 4,094 (1.79%) patients received respectively TKA, THA, and MJA under the diagnosis of RA (Figure 1).

### General Population

**Figure 2:**
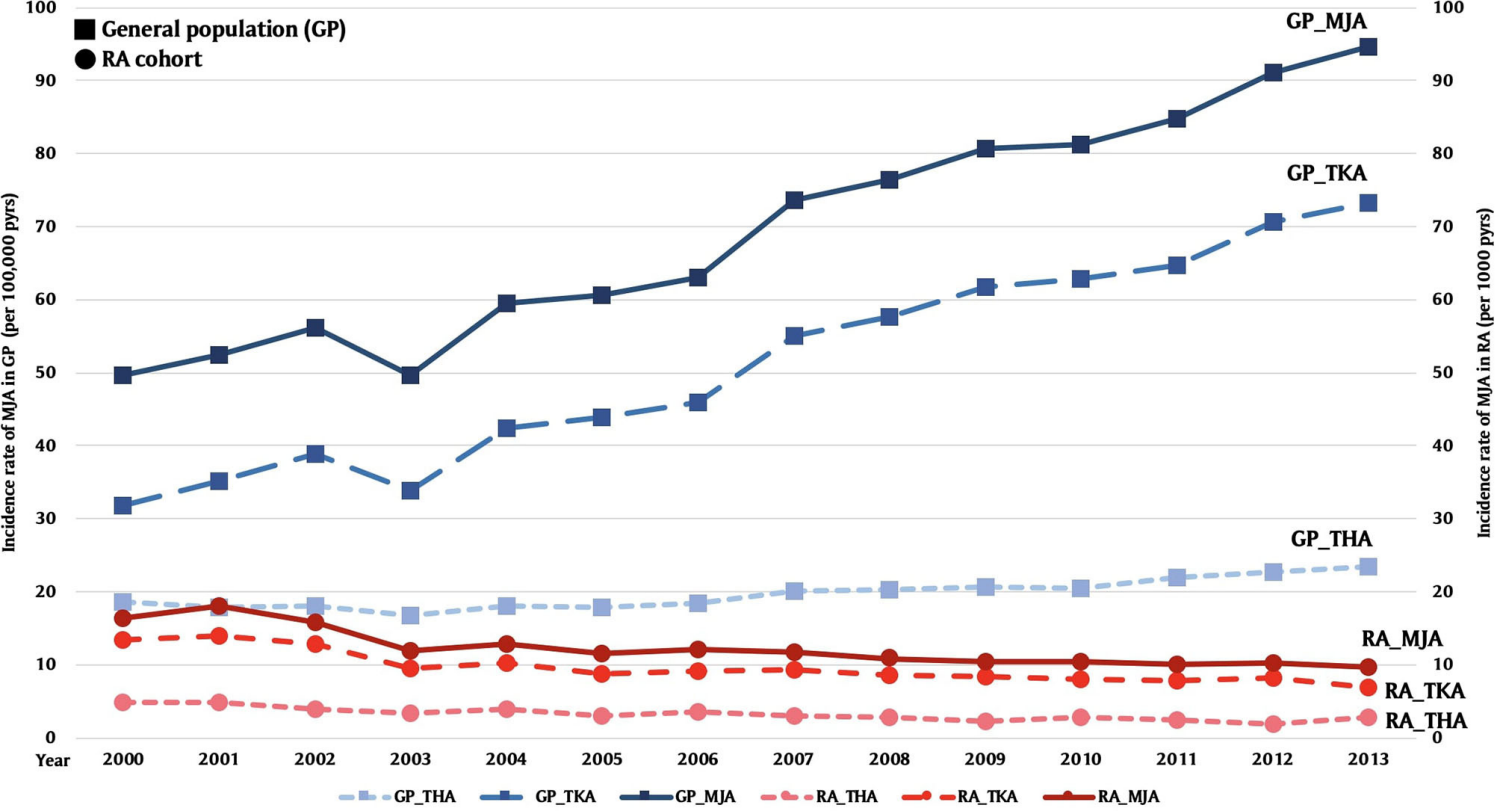
Comparison of incidence rates of total hip arthroplasty (THA), total knee arthroplasty (TKA), and major joint arthroplasty (MJA) between general hospitalization patients (GP) and rheumatoid arthritis (RA) patients from 2000 to 2013 [per 100,000 person years (pyrs) in GP and per 1,000 pyrs in RA patients].

Among the GP, comparing within years from 2000 to 2013, we found significant increments in their number of surgery and incidence rate (*p* for trend < 0.001): for TKA (*N* = 7,068–17,432 with incidence rate from 31.70 to 73.30 per 100,000 person years), THA (*N* = 4,160–5,583 with incidence rate from 16.64 to 23.39 per 100,000 person years), and MJA (*N* = 11,075–22,438 with incidence rate from 49.70 to 94.56 per 100,000 person years) (Figure 2). The ratio of incidence rate between 2013 and 2000 was 2.31 for TKA (95% CI 2.25–2.38), 1.25 for THA (95% CI 1.20–1.31), and 1.90 for MJA (95% CI 1.86–1.95). Patients aged between 70 and 79 years were the dominant population receiving TKA, THA, or MJA surgeries. Females generally received one to three times more TKA, THA and MJA surgeries than males over 60 years of age.

### Patients With RA

**Figure 3:**
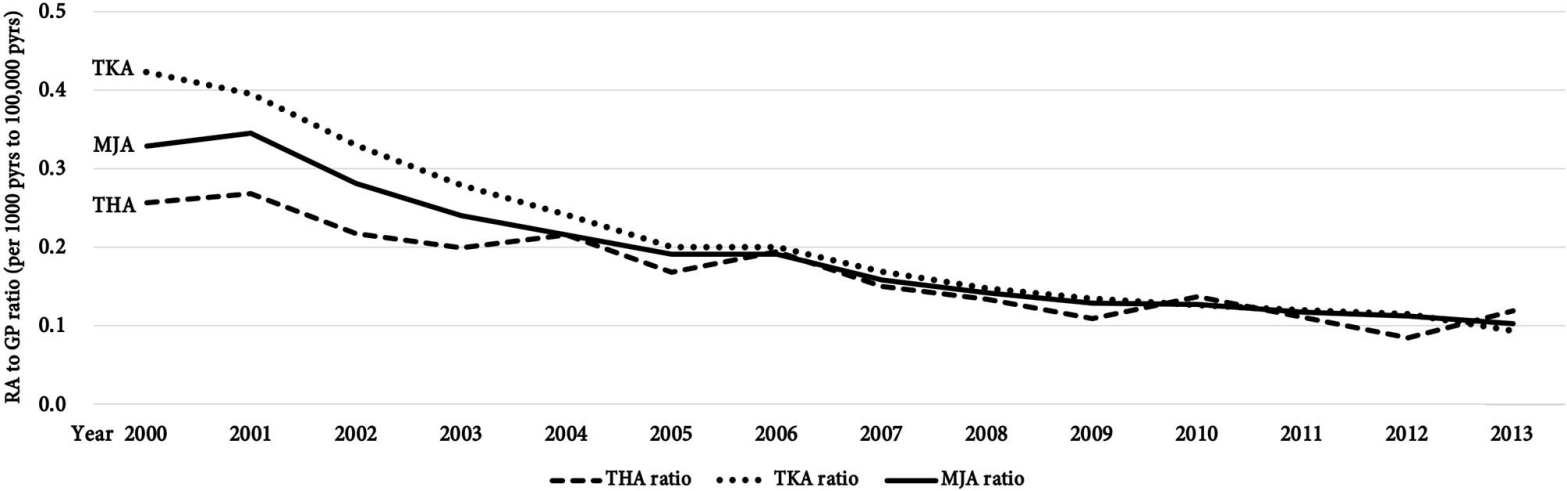
Incidence ratio of rheumatoid arthritis (RA) patients to general hospitalization patients (GP) from 2000 to 2013 [per 100,000 person years (pyrs) in GP and per 1,000 pyrs in RA patients], indicating the proportion of RA patients receiving total hip arthroplasty (THA), total knee arthroplasty (TKA), and major joint arthroplasty (MJA) among GP cohort.

Of the 35,219 patients among RA cohort, 76.9% were women, and the mean age was 53.7 ± 13.8 of the RA cohort (Table 1). Most of the patients were office workers. Among RA patients, 1,421 (4.0%) had osteoarthrosis, 317 (0.9%) had Sjogren syndrome, 1,021 (2.9%) had gout, 211 (0.6%) had systemic lupus erythematous, 154 (0.4%) had ankylosing spondylitis. Patients of 70–79 years old were the dominant age group receiving TKA, THA, or MJA surgery. Regarding gender differences, females over age 60 years received more TKA, THA, and MJA than males. Females over 40 and 60 years-old also had higher incidences in receiving TKA and THA than males (Table 2). We further verified the association between comorbidities and TKA, THA, and MJA among RA cohort (Table 3). Sjogren syndrome (0.34, 95% CI 0.13–0.9) and osteoporosis (0.34, 95% CI 0.14–0.83) were less likely to receive TKA whilst coexisting with osteoarthrosis (2.59, 95% CI 2.23–3.01) increased the risk; ankylosing spondylitis (4.8, 95% CI 2.85–8.10) and osteoarthrosis (1.72, 95% CI 1.21–2.43) are associated with higher relative risk in receiving THA; osteoporosis (0.44, 95% CI 0.21–0.94) was less likely to receive MJA whilst osteoarthrosis (2.46, 95% CI 2.14–2.84) and ankylosing spondylitis (1.87, 95% CI 1.19–2.95) increased the risk. Obesity showed no impact to the relative risk for receiving surgeries. Comparing within years from 2000 to 2013, we found significant drop in numbers undergoing surgery numbers and incidence rate (*p* for trend < 0.001): for TKA (*N* = 176–270 with incidence rate from 13.42 to 6.85 per 1,000 person years), for THA (*N* = 65–113 with incidence rate from 4.79 to 2.80 per 1,000 person years), and for MJA (*N* = 210–347 with incidence rate from 16.36 to 9.65 per 1,000 person years) (Figure 2). Incidence rate ratios between year 2013 and 2000 were 0.51 for TKA (95% CI 0.42–0.62), 0.58 for THA (95% CI 0.43–0.79), and 0.59 for MJA (95% CI 0.5–0.7) (Table 4). Apparent decreasing incidence ratio of TKA, THA, and MJA of RA to GP cohort from 2000 to 2013 are demonstrated in Figure 3.

**Table 1:** Demographic characteristics and comorbidities of patients newly diagnosed rheumatoid arthritis (RA) in Taiwan from 2000 to 2013.

**Variable**	**RA Patients (*n* = 35,219)**
	***n* (%)/mean (SD)**
**Gender**	
Female	27,096 (76.9)
Male	8,123 (23.1)
**Age**	
20–39	5,800 (16.5)
40–64	21,753 (61.8)
≥65	7,666 (21.8)
Mean (SD)	53.7 (13.8)
**Occupation**	
Office workers	17,806 (50.6)
Manual workers	14,672 (41.7)
Others	2,741 (7.8)
**Baseline comorbidity**	
Hypertension	3,501 (9.9)
Diabetes mellitus	1,992 (5.7)
Chronic liver disease	1,673 (4.8)
Osteoarthrosis	1,421 (4.0)
Gout	1,021 (2.9)
Hyperlipidemia	987 (2.8)
COPD	753 (2.1)
Sjogren syndrome	317 (0.9)
Systemic lupus erythematous	211 (0.6)
Ankylosing spondylitis	154 (0.4)
Osteoporosis	148 (0.4)
Obesity	38 (0.1)

*COPD, chronic obstructive pulmonary disease; RA, rheumatoid arthritis; SD, standard deviation*.

**Table 2:** Incidence rate and number of cases stratified by gender and age groups among rheumatoid arthritis (RA) patients from 2000 to 2013.

**TKA**							
**Age group**	**Total**		**F**		**M**		**F/M ratio**
	***N***	**IR**	***N***	**IR**	***N***	**IR**	
**Overall**	3,418	4.23	2,862	4.55	556	3.12	1.46
20–29	33	0.65	25	0.62	8	0.76	0.81
30–39	98	0.88	78	0.87	20	0.94	0.92
40–49	284	1.47	247	1.60	37	0.96	1.66
50–59	927	4.51	817	5.04	110	2.53	1.99
60–69	1252	8.32	1057	9.31	195	5.29	1.76
70–79	723	9.33	571	10.30	152	6.90	1.49
80–100	101	5.40	67	5.08	34	6.16	0.82
**THA**							
**Overall**	1,159	1.37	859	1.29	300	1.65	0.78
20–29	54	1.07	29	0.72	25	2.44	0.29
30–39	88	0.79	61	0.68	27	1.29	0.52
40–49	174	0.89	120	0.77	54	1.42	0.54
50–59	309	1.45	237	1.40	72	1.63	0.86
60–69	308	1.87	242	1.92	66	1.71	1.12
70–79	203	2.28	148	2.27	55	2.32	0.98
80–100	23	1.07	22	1.42	1	0.17	8.57
**MJA**							
**Overall**	4,079	10.75	3,322	10.96	757	9.93	1.10
20–29	72	5.02	43	4.15	29	7.31	0.57
30–39	165	4.93	125	4.44	40	7.52	0.59
40–49	402	5.57	318	5.27	84	7.07	0.75
50–59	1089	10.27	934	10.71	155	8.23	1.30
60–69	1385	15.86	1160	16.85	225	12.18	1.38
70–79	833	16.18	647	17.00	186	13.86	1.23
80–100	117	7.96	82	7.87	35	8.16	0.97

*IR, Incidence rate; F, female; M, male; N, number of cases*.

* *Incidence rate:per 1,000 person years*.

**Table 3:** Cox model measured hazard ratio and 95% confidence intervals of TKA, THA, or MJA associated with and without comorbidities among rheumatoid arthritis (RA) patients.

	**TKA**		**THA**		**MJA**	
**Characteristics**	**aHR (95% CI)**	***p*-value**	**aHR (95% CI)**	***p*-value**	**aHR (95% CI)**	***p*-value**
**Baseline comorbidity**						
Hypertension	1.04 (0.89–1.21)	0.620	0.97 (0.69–1.35)	0.839	1.04 (0.90–1.19)	0.629
Diabetes mellitus	1.06 (0.88–1.28)	0.569	0.77 (0.50–1.20)	0.246	1.04 (0.87–1.23)	0.687
Hyperlipidemia	1.02 (0.79–1.31)	0.899	0.90 (0.52–1.57)	0.719	1.00 (0.79–1.27)	0.993
COPD	0.72 (0.51–1.01)	0.053	0.89 (0.48–1.64)	0.699	0.74 (0.55–1.00)	0.051
Chronic liver disease	1.00 (0.81–1.23)	0.984	1.40 (0.98–2.00)	0.064	1.06 (0.88–1.27)	0.560
Gout	1.03 (0.80–1.33)	0.812	1.41 (0.93–2.13)	0.102	1.13 (0.91–1.41)	0.280
Sjogren syndrome	0.34 (0.13–0.90)	0.029	1.13 (0.43–3.09)	0.777	0.54 (0.27–1.09)	0.085
SLE	1.22 (0.74–1.99)	0.438	1.76 (0.79–3.94)	0.169	1.42 (0.93–2.16)	0.105
Ankylosing spondylitis	0.49 (0.18–1.30)	0.149	4.80 (2.85–8.10)	<0.001	1.88 (1.19–2.95)	0.007
Osteoarthrosis	2.59 (2.23–3.01)	<0.001	1.72 (1.21–2.43)	0.003	2.46 (2.14–2.84)	<0.001
Osteoporosis	0.34 (0.14–0.83)	0.017	1.16 (0.29–4.71)	0.832	0.44 (0.21–0.94)	0.033
Obesity	–	–	–	–	–	–

*HR, hazard ratio; CI, confidence interval; SLE, systemic lupus erythematous*.

*aHR: HR was adjusted for gender, age, occupation and all comorbidities in Cox proportional hazards regression*.

**Table 4:** Annual numbers and incidence rates [per 1,000 person years (pyrs)] compared to year 2,000 among patients with rheumatoid arthritis receiving total hip arthroplasty (THA), total knee arthroplasty (TKA), and major joint arthroplasty (MJA), respectively.

	**THA**				**TKA**				**MJA**			
**Year**	***N***	**Total population person years**	**Annual incidence rate**	***P*-value**	***N***	**Total population person years**	**Annual incidence rate**	***P*-value**	***N***	**Total population person years**	**Annual incidence rate**	***P*-value**
2000	65	13,560	4.79 (3.70–6.11)		176	13,111	13.42 (11.51–15.56)		210	12,837	16.36 (14.22–18.73)	
2001	76	15,829	4.80 (3.78–6.01)	0.992	213	15,331	13.89 (12.09–15.89)	0.736	268	14,797	18.11 (16.01–20.42)	0.270
2002	71	18,070	3.93 (3.07–4.96)	0.247	224	17,528	12.78 (11.16–14.57)	0.625	264	16,731	15.78 (13.93–17.80)	0.696
2003	68	20,501	3.32 (2.58–4.20)	0.034	188	19,916	9.44 (8.14–10.89)	0.001	225	18,889	11.91 (10.41–13.57)	0.001
2004	90	22,962	3.92 (3.15–4.82)	0.216	229	22,327	10.26 (8.97–11.68)	0.007	270	21,051	12.83 (11.34–14.45)	0.008
2005	76	25,154	3.02 (2.38–3.78)	0.006	215	24,472	8.79 (7.65–10.04)	<0.001	265	22,954	11.55 (10.20–13.02)	<0.001
2006	98	27,416	3.57 (2.90–4.36)	0.067	245	26,693	9.18 (8.07–10.40)	<0.001	300	24,931	12.03 (10.71–13.48)	0.001
2007	89	29,515	3.02 (2.42–3.71)	0.005	267	28,751	9.29 (8.21–10.47)	<0.001	312	26,724	11.68 (10.42–13.05)	0.000
2008	86	31,544	2.73 (2.18–3.37)	0.001	261	30,759	8.49 (7.49–9.58)	<0.001	310	28,489	10.88 (9.70–12.16)	<0.001
2009	76	33,503	2.27 (1.79–2.84)	<0.001	272	32,672	8.33 (7.37–9.38)	<0.001	314	30,156	10.41 (9.29–11.63)	<0.001
2010	99	35,319	2.80 (2.28–3.41)	0.001	275	34,425	7.99 (7.07–8.99)	<0.001	328	31,662	10.36 (9.27–11.54)	<0.001
2011	91	36,963	2.46 (1.98–3.02)	<0.001	280	36,033	7.77 (6.89–8.74)	<0.001	329	33,049	9.96 (8.91–11.09)	<0.001
2012	74	38,742	1.91 (1.50–2.40)	<0.001	308	37,776	8.15 (7.27–9.12)	<0.001	352	34,552	10.19 (9.15–11.31)	<0.001
2013	113	40,379	2.80 (2.31–3.36)	0.001	270	39,399	6.85 (6.06–7.72)	<0.001	347	35,959	9.65 (8.66–10.72)	<0.001

*P-values < 0.05 are statistically significant*.

## Discussion

We found significantly lower incidence rates and numbers of TKA, THA, and MJA among the patients with RA, in contrast to a concomitantly rising trend in the general hospitalized population during the study period from 2000 to 2013. Opposing trend of incidence in total joint arthroplasty was also reported in several studies (11, 16, 17). However, comparison focusing on MJA among RA patients in Asian population has not been addressed. Our present study is the first population-based nationwide analysis based on the NHIRD records to reveal the opposing incidence trends over a period of 14 years in TKA, THA, and MJA between well-defined RA patients and general hospitalized population in Taiwan.

Our major results were the incidence of TKA, THA, and MJA among GP significantly increased by 131, 25.1, and 90% over 14 years, respectively (Figure 2). This rising trend is in line with previous epidemiology observations in Taiwan and worldwide (14, 18, 19). Noted that the decline of incidence in both TKA and THA in year 2003 we found could be attributed to the pandemic of severe acute respiratory syndrome (SARS) which prevented patients from undergoing TKA and THA (20). On the other hand, we found significant drops in the incidence rate of TKA, THA, and MJA among patients with RA by 49.0, 41.5, and 41.0%, respectively (Figure 2). According to the above findings, the proportion of RA patients receiving joint replacements among GP showed apparent decline trend by dividing incidence of GP by that of RA cohort (Figure 3). The falling trend is consistent with previous studies in both Europe (11, 12, 16, 17, 21–24) and the USA (9, 10, 25), whereas few reports have been published on Asian countries. These regional- and national-scale database studies provided enough power for the reliability of such findings. Comparison between reports related to incidence changes in joint arthroplasty surgery among RA patients is shown in Table 5. Since 1955, total joint arthroplasty surgeries (TJR), including total ankle, elbow, hip, shoulder, and knee showed a declining trend after the trajectory plateau of incidence (11, 12, 26). The difference in surgical incidences between our present study and the literature could be due to the heterogeneity of disease, alternative health insurance system, and intensity of treatment strategies. Females tended to receive more TKA and THA than males during the 4th−7th decades of their lives, a finding which is consistent with the age-specific incidence of RA as previously reported (27).

**Table 5:** Comparison of recent studies targeting incidences of joint arthroplasty surgeries among patients with rheumatoid arthritis.

**References**	**Numbers**	**Country**	**Change in incidence of joint arthroplasties**	**Study period**
da Silva et al. (9)	609	The USA	TKA and THA↓	1955–1995
Weiss et al. (22)	159,888	Sweden	TJA↓	1987–2001
Fevang et al. (17)	8,268	Norway	THA, and TJA↓ in chronic inflammatory joint disease (RA included) TKA: stable	1994–2004
Sokka et al. (26)	263,869	Finland	No change in TKA and THA	1986–2003
Louie and Ward (10)	22,055 (TKA) 16,529 (THA)	The USA	TKA and THA↓ in age 40–59 But TKA and THA↑ in age ≥60	1983–2007
Hekmat et al. (23)	2,164	Malmö, Sweden	THA↓ TKA showed stable	1998–2007
Skyttä et al. (16)	120,787	Finland	TKA↓ whilst GP↑	1980–2010
Jämsen et al. (11)	245,854	Finland	TJA↓ (including TKA and THA)	1995–2010
Mertelsmann-Voss et al. (25)	2,839,325	The USA	TKA and THA↓ in age>40	1991–2005
Hawley et al. (30)	17,505	UK	TKA↓ in TNFi cohort THA showed stable	1995–2014
Cordtz et al. (31)	297,916	Denmark	TKA and THA↓ whilst GP↑	1996–2001
Young et al. (12)	8,690,061	The USA	TJA, TKA, THA↑	2002–2012
Hawley et al. (24)	19,116	UK	THA↓ in age ≥ 60	1995–2014
Hawley et al. (21)	27,607	UK	TKA and THA showed stable	1995–2014
Current study	228,191 (MJA) 168,457 (TKA) 64,543 (THA)	Taiwan	TKA, THA, and MJA↓	2000–2013

*↑, increase of incidence; ↓, decrease of incidence; GP, general populations; THA, total hip arthroplasty; TJA, total joint arthroplasty, including total ankle, elbow, hip, knee, and shoulder arthroplasty; TKA, total knee arthroplasty; MJA, major joint arthroplasty, including TKA and THA; RA, rheumatoid arthritis*.

RA in Taiwan has increased steadily in the last decade, with incidence rising from 14.2 to 15.2 per 100,000 people, and prevalence from 74.5 to 118.3 cases per 100,000 people (28). These patients have 4 times higher risk of undergoing TKA, THA, and MJA surgeries comparing with non-RA patients (6). Moreover, arthroplasty covered by Taiwan NHI program has also extended the indications and improved access for TKA and THA (18, 29). It is therefore reasonable to assume that demands for RA-related orthopedic procedures, including the need for MJA, might have increased with time along the escalated late-phase RA population. However, our current results showing a decline in demands of TKA, THA and MJA surgeries, are indirect support of the improving disease outcomes. Early observations from the Rochester Epidemiology Project showed that RA patients diagnosed after year 1985 were less likely to require total joint arthroplasty, matching the more frequent use of conventional DMARDs, especially methotrexate, hydroxychloroquine, and sulfasalazine (9, 11, 16). Introduction and aggressive prescriptions of biological DMARDs such as tumor necrosis factor inhibitor (TNFi) have lowered MJA rates since 2001 (23, 30, 31). In Taiwan, the more frequent use of DMARDs, especially after approving the prescription of adalimumab and etanercept in 2002, matched the concomitant drops in TKA, THA, and MJA surgeries in our RA patients (32–34).

Moreover, the fact that lower healthcare utilization cost was offset with the increase medication cost in biologics use may be alleviated by improvement in outcomes of RA in Taiwan since 1990s (5). Such constant occurrence is also linked with the improving long-term outcomes. Using the US Nationwide Inpatient Sample, significantly lower prevalence were found with total shoulder and elbow arthroplasty whereas prevalence of TKA and THA remained unchanged (12). In Finland, such constant occurrence of MJA was reported with RA patients, even before the emergence of biological DMARDs. At the same time an increase of MJA at 2–10-fold was found with the non-RA population (26). Similar occurrence of MJA was found in England from 1995 to 2004 (21). However, a relative 34% drop in incidence of TKA but not of THA was associated with the introduction of TNFi among a similar cohort (30). Supported by previous observations, the potential influence of modern pharmacological treatment strategies has been considered to be the plausible cause for the control of moderate to severe RA progression, and indirectly decreased or maintained the incidence of MJA surgery conducted worldwide. Moreover, patients seeking medical care toward milder cases, early detection of RA according to adjustment of criteria, and general improvement of RA disease may also contribute to such a decline (35, 36).

Meanwhile, the increasing trends of TKA, THA, and MJA in the GP could be explained by the rising number of symptomatic osteoarthrosis (OA) (96.9%) and avascular necrosis (46.9%), which were accountable for major contributors toward TKA and THA. RA was accountable for only 1–3% in TKA, and 0.5% in THA (29, 37). The rising elderly population and overweight individuals both increase demands for TKA with advanced OA. During the period from 2002 to 2010, the age group > 65 year-old accounted for 9–10.7% of the population, at an obesity rate of 17–18% (18, 29). Symptomatic OA is often caused by articular degeneration such as wearing and progressive loss of joint cartilage. Unlike OA, synovitis in RA often results from the destruction of joint cartilage, bone density loss, and periarticular osteopenia. Both destructive mechanisms end in irreversible joint deformity which requires arthroplasty. The current study shows patients with the combination of OA and RA have a 1.7–3.6 times higher risk to receive TKA, THA, and MJA. It has been reported that the risk of developing RA is higher in patients with symptomatic OA and OA-related surgery based on the similar expression of proinflammatory and anti-inflammatory cytokines. The synergistic effect of the disease may lead to higher demands of arthroplasties (38). However, these patients were included in the RA cohort according to our study design, and they underwent fewer TKA, THA, and MJA surgeries.

Patients with osteoporosis in RA have lower risks for receiving TKA and MJA in current study. Osteoporosis in RA, caused by rapid remodeling of bone, is associated with joint instability and disease activity. Unlike postmenopausal osteoporosis, radius and hip joint bone were mainly affected whilst axial bone were relatively preserved in RA-related osteoporosis (39). However, patients undergoing elective MJA were lack of identification and treatment for osteoporosis (40). Actual fracture risk, disease activity, and glucocorticoid dosage should be well-evaluated for clinical practicing. We also found significant higher risk in RA patients receiving THA and MJA whilst coexist with ankylosing spondylitis (AS), since the hip joint was the most commonly effected peripheral joint of the inflammatory process (41). However, the co-existence of AS and RA is relatively uncommon. A proportion of these patients may have had seronegative RA/AS predominant phenotype which accounts for the significant differences in hip joint replacement data. On the other hand, primary Sjogren's arthritis are rarely damaging, whilst second hit of RA may be the leading cause in receiving MJA (42). SLE/RA overlap is not uncommon (43). However, it may be difficult to use the data with higher potential of anti-inflammatory agent control in this group (44). BMI data (including heights or weights) were not included in the NHIRD, thus, we replaced BMI with the diagnosis of Obesity (ICD-9-CM: 278 and A183). However, it is noted that obesity showed no impact on the relative risk in receiving TKA, THA, and MJA. This result should be cautiously interpreted since the small patient number may cause a relatively low impact on the regression model. Therefore, while rising number and incidence rate among GP provided greater operating capacity for TKA, THA, and MJA, an opposite trend of decline among RA patients indirectly supported the pharmacological potential on improving clinical status of the disease.

Below we describe the limitations and merits of our present study. First, the methodological limitations complicated our deduction for the impact of medication on RA patients that resulted in a drop of TKA, THA, and MJA, due to our retrospective design. The indications, the usage of different anti-RA drugs, and different disease severity of patients have probably confounded the results. Moreover, alternative health care systems were difficult to compare with each other. Second, RA incidences could have been underestimated. Since those with milder RA may have not applied for the catastrophic disease registry in the NHIRD. Moreover, the lag period between onset of RA symptoms and the diagnosis of the disease may require cautious interpretation for the time-specific cumulative incidence. The incidences TKA, THA, and MJA may have been miscalculated due to RA may not have been the primary indication for the surgery. However, the inflammatory process of RA contributed greatly to joint destruction, leading to inevitable joint arthroplasty surgeries. It is difficult to discriminate influences between comorbidities such as OA from RA due to high rates of incidence and prevalence in the elderly. The introduction of ACR 2010 criteria (36) may have altered incidence rates of surgery due to earlier diagnoses of RA. Longer term follow-up is needed for the evaluation of such bias due to RA and comorbidities were chronic diseases.

Our present interrupted time-series analysis has several advantages. We matched the incidence of TKA, THA, and MJA to clarify the possible impact of the opposite trend between GP and patients with RA from a well-collected national-wide population-based database. To specifically address the impact of anti-RA drugs on RA patients, the design with random control trials is difficult. Thus, we chose to study on a high credibility retrospective cohort based on a large-scale database. The national burden of RA can now be better handled based on what we found on incidence trends of MJA and the possible effectiveness of pharmacological treatments. MJA surgeries are highly utilized procedures with heavy load on the NHI resources in Taiwan. The increasing trend of MJA among the GP noted in our current study suggested the significant impact on health insurance expenditure in the future. However, the concomitant decreasing trend in MJA among RA patients allowed authority to focus the budget planning on effectiveness early aggressive treatment strategies, not MJA, among patients with RA.

## Conclusion

We have demonstrated a substantial increase in incidences of TKA, THA, and MJA among the general population with a concomitant drop in RA patients during a 14-year period from 2000 to 2013 in Taiwan. Opposite trends for surgical incidences were provided for comparison across Asian populations. The potential influence of pharmacological treatment is a plausible cause for the lowered incidence of MJA surgeries, and the improvement in disease condition among RA patients. With cautious interpretation of our current findings, cost-effectiveness assessment of strategies for RA treatment could be conducted for reducing the financial burden of our national health insurance program.

## Data Availability Statement

The original contributions presented in the study are included in the article/supplementary material, further inquiries can be directed to the corresponding author/s.

## Ethics Statement

The Research Ethics Committee of China Medical University and Hospital, Taiwan approved our study [CMUH-104-REC2-115-(AR4)].

## Author's Note

At the time of submission, this is the first report to discuss the opposing incidence trends of the total knee arthroplasty, total hip arthroplasty, and major joint arthroplasty between well-defined patients with rheumatoid arthritis (RA) and general hospitalized population in Taiwan and Asia.

This report is interesting for the reason that despite the effectiveness of pharmacological treatment, orthopedic procedures still possess a crucial role in management of severe RA. However, increased intensity of more effective anti-rheumatic medications has been proposed to be associated with decreased incidence of procedures, specifically joint arthroplasty surgeries.

Overall, we believe that current findings of opposing trend of joint arthroplasty surgeries provide greater insights into epidemiology observation in effectiveness in pharmacological treatment of RA among Asian populations.

## Patient and Public Involvement

All data were extracted from Taiwan National Health Insurance Research Database. Patients and the public were not involved.

## Author Contributions

K-KT, M-CL, and JW had full access to all of the data in the study and take responsibility for the integrity of the data and the accuracy of the data analysis. K-KT, M-CL, and JW: study concept and design, acquisition of data. M-CL: statistical analysis. K-KT, Y-HL, C-HL, and C-CL: drafting of the manuscript. JW: study supervision. All authors: interpretation of data and critical revision of the manuscript for important intellectual content.

## Conflict of Interest

The authors declare that the research was conducted in the absence of any commercial or financial relationships that could be construed as a potential conflict of interest.
